# Safety and efficacy of therapeutic taping in primary dysmenorrhea: a systematic review and meta-analysis

**DOI:** 10.1038/s41598-022-11034-w

**Published:** 2022-05-03

**Authors:** E. M. I. A. Bandara, W. N. I. Kularathne, K. Brain, Ishanka Weerasekara

**Affiliations:** 1 Department of Physiotherapy, Faculty of Allied Health Sciences, University of Peradeniya, Peradeniya, 20400 Sri Lanka; 2 Human Health Sciences, Graduate School of Medicine, Kyoto University, Kyoto, Japan; 3 National Hospital, Kandy, 20000 Sri Lanka; 4 School of Health Sciences, College of Health, Medicine and Wellbeing, The University of Newcastle, Callaghan, Australia

**Keywords:** Diseases, Health care, Medical research

## Abstract

Primary dysmenorrhea (PD) is a common gynecological condition among adolescent and adult women. Several pharmacological and alternative therapies (e.g. therapeutic taping) have been used to treat PD, with varying effect. This systematic review and meta-analysis was performed to evaluate the safety and efficacy of therapeutic taping on clinical symptoms of PD, considering pain as the primary outcome. MEDLINE, Cochrane Library, Embase, PEDro, CINAHL and gray literature sources were searched from inception to February 2022 for randomized controlled trials (RCTs) that assessed the effect of therapeutic taping for PD. The language was restricted to English. A total of ten studies were included in the systematic review, involving 685 participants. Eight studies were included in quantitative analysis. The quality of the studies ranged from 4 to 7 with a median of 5 as assessed by PEDro scale. Meta-analyses indicated short-term improvements of pain compared to sham and no interventions. Elastic therapeutic taping (ETT) indicated short term improvements in anxiety associated with PD. Moderate to high quality of evidence suggested that ETT is an effective intervention in improving pain, anxiety, and quality of life of women with PD. A scarcity of evidence on the long-term effects of therapeutic taping in PD is observed.

## Background

Dysmenorrhea is a painful and cramping sensation that occurs in the lower abdominal area accompanied by other features, such as backpain, nausea, vomiting, sweating, dizziness, headache, diarrhea, and tiredness^1^. These symptoms usually occur a few days before or during menstruation^1,2^. The burden of dysmenorrhea is significantly higher than any other gynecological complaint^3^. It is a prominent cause of gynecological morbidity in females of reproductive age^4,5^. Prevalence is high, with 45–93% of women in their reproductive age experiencing dysmenorrhea, and the highest rates are reported in adolescent girls^3,6^. Depending on the pathophysiology, dysmenorrhea can be divided into two categories; primary and secondary^7^. Primary dysmenorrhea (PD) is described as a cramping pain in the lower abdomen which occurs without any obvious pelvic pathology^1^. Secondary dysmenorrhea is described as the menstrual pain caused by underlying pelvic pathology, such as endometriosis, adenomyosis, intra uterine adhesions, cervical stenosis, ovarian cysts, uterine myomas or polyps, infertility problems and pelvic inflammatory disease. The onset may be years after first menstruation^8,9^. PD often occurs in women who are under 20 years of age, after menarche^1^. The exact cause of PD is not well identified. However, it is hypothesized that excessive production of uterine prostaglandins, particularly of prostaglandin F2alpha (PGF2a) and prostaglandin F2alpha (PGF2) is involved in the pathogenesis of PD^10^. Excessive uterine prostaglandin levels increase uterine tone and high amplitude contractions^10^. Several risk factors for PD have been identified; age (< 20 years), smoking, nulliparity, longer and heavy menstrual flow, high body mass index (BMI), earlier onset of menarche, family history, depression, anxiety and stress^11,12^. Women experiencing PD often report poor physical, mental and social wellbeing. Poor academic performance, absenteeism from school and work, limitations of daily activities, poor quality of sleep, increased levels of stress, anxiety and depression are examples of the reported consequences of PD^1,4,8^. Consequences not only affect women on an individual level, but they also impact the community and economy with decreased productivity leading to economic loss^1,4^. However, most women do not report or seek medical attention for PD, because it is considered a normal feature of menstruation^13^.

Both pharmacological and nonpharmacological treatments are available to manage PD^14^, while surgical procedures are also available for extreme cases^15^. Pharmacological treatments target the physiological mechanisms associated with menstrual pain and other symptoms. Aspirin, paracetamol and nonsteroidal anti-inflammatory drugs (NSAIDs) are believed to reduce the activity of cyclo-oxgenase pathways, thus inhibiting excessive production of prostaglandins. Oral contraceptives are also used to inhibit ovulation. A combination of analgesics and oral contraceptives may be useful in cases where women do not respond to a single treatment^7^. There are some side-effects associated with analgesics and contraceptives. Gastrointestinal disturbances (nausea, vomiting and diarrhea) were observed with the use of NSAIDS. Side-effects such as nausea, abdominal pain, headache, acne, bloating, anxiety, loneliness and weight gain were reported with the use of oral contraceptives^7,16^. Approximately, 10–20% of women do not respond to pharmacological management and some may have contraindications for the use of NSAIDs and oral contraceptives^7^. Furthermore, there is emerging evidence that suggests serotonin-based pharmacotherapy (i.e. melatonin-fluoxetine combination and antidepressants) may be an alternative treatment to hormone replacement therapy for dysmenorrhea and menopausal symptoms^17,18^. Alternative treatments play an important role in management dysmenorrhea. Evidence shows that patients have greater satisfaction and reduced medication intake and pain when using some alternative therapies^19–21^. Common alternative therapies include herbs such as aloe vera, chamomile, cinnamon, fennel, and ginger^22^, dietary changes such as a low-fat vegetarian diet, vitamins (B, C, E), and supplements (calcium and magnesium)^23–25^, hypnosis and psychotherapy^24,25^. Physiotherapy treatments also play a role in managing PD. Generally, physiotherapy treatments for PD include heat therapies, exercises, relaxation therapies, connective tissue massage, acupressure, acupuncture, Transcutaneous Electrical Nerve Stimulation (TENS), spinal manipulation and taping, such as kinesiotaping (KT) and elastro-tapes^25,26^.

**Figure 1:**
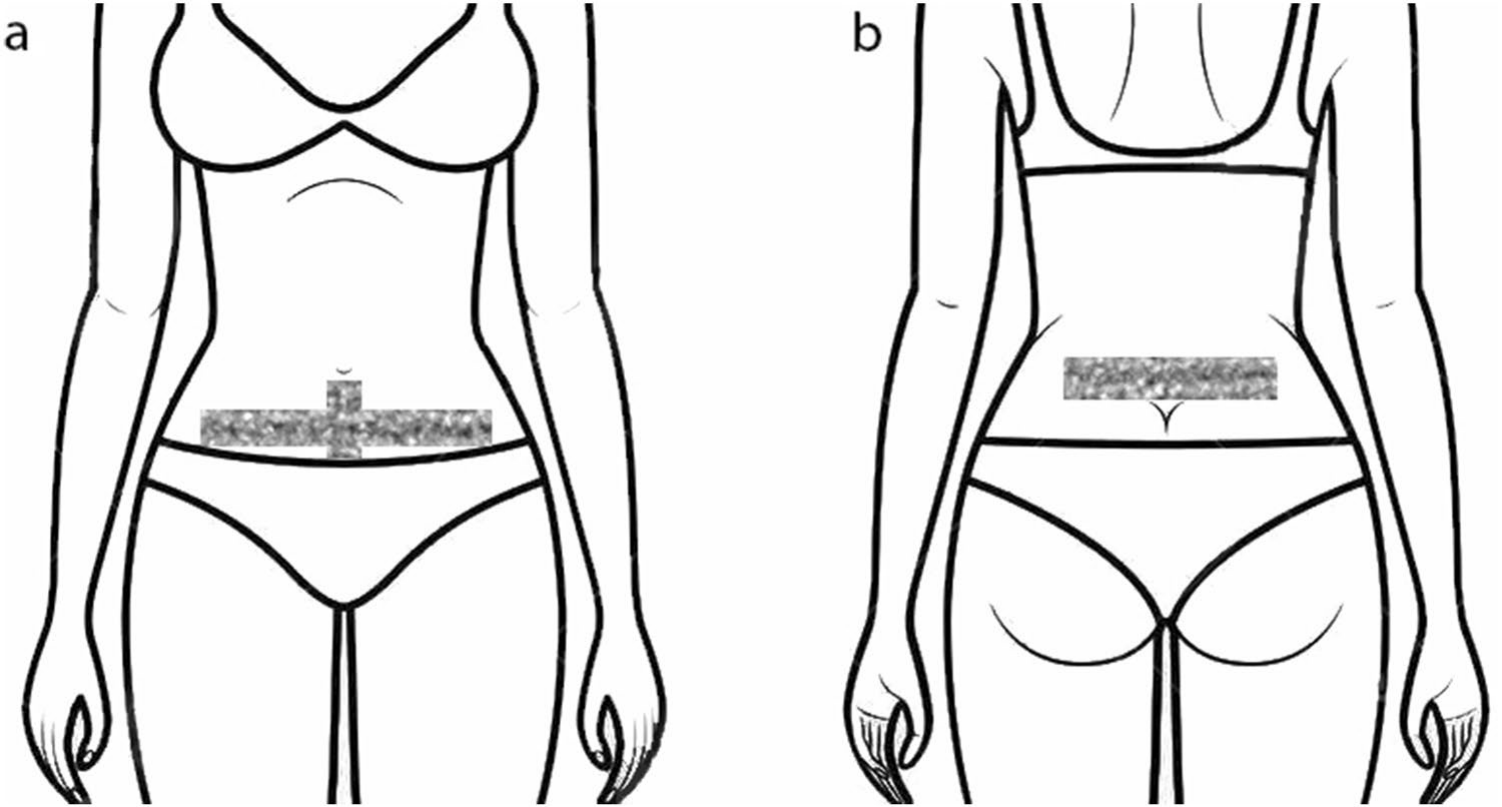
Schematic illustration of therapeutic tape application for PD. (**a**) front view (**b**) back view.

There are a variety of tapes available, each with different types, materials and uses. Common tapes are rigid tapes (a non-elastic and non-permeable), KT, and spiral tapes (a non-elastic synthetic tape applied in a spiral or grid shape)^27^. Spiral tapes may generate cutaneous stimuli, reduce pain and swelling, improve circulation and regulate muscle tone and metabolism^28^. KT is a specially designed elastic tape designed to maintain air permeability, be water-resistant and contain hypoallergic materials. This taping type used widely in clinically settings as it has a strong adherence, low risk of skin irritation, long lasting capacity and is easy to apply. KT is found to be effective in reducing pain, supplying proprioceptive feedback, stimulating muscle activity, supporting weak muscles, and increasing lymphatic and blood flow to the applied area^29,30^ (Fig. 1).

Several research studies have investigated the effectiveness of taping for PD. However, most of the RCTs have been conducted with small sample sizes. Inconsistencies were observed among the findings from previous RCTs as some studies favor therapeutic taping for pain management in PD^31,32^ while others find no significant superiority compared to placebo application^19^. Lack of high-quality evidence in this field has informed this systematic review and meta-analysis which collates evidence aiming to determine whether taping is an effective and safe treatment for PD in improving pain and related clinical symptoms, such as anxiety.

## Methods

This systematic review and meta-analysis was performed following the Preferred Reporting Items for Systematic Reviews and Meta-Analyses (PRISMA) guidelines^33^. The study protocol for this systematic review was registered with International Prospective Register of Systematic Reviews (PROSPERO) on 24th June 2021 (CRD42021256578).

### Eligibility criteria

RCTs published in English were included if they assessed women of any age with PD. Study participants had to be treated with a therapeutic tape application (either alone or in combination with another therapy) aiming to treat pain associated with PD. Therapeutic tape applications were varied, such as elastic therapeutic tape (ETT), rigid therapeutic tape (RTT) and spiral tape. Studies with other therapeutic interventions, sham taping or no intervention control groups were included. Studies also had to include a measure of pain to be considered eligible for inclusion. Studies which satisfied any of the following criteria were excluded; study protocols, abstract-only papers (eg: proceeding papers, conference abstracts, editorials, and commentaries), and when the full text was not available.

### Search strategy

The following databases were searched; CINAHL, Cochrane Library, Embase, MEDLINE and PEDro (Physiotherapy Evidence Database); with keywords related to therapeutic taping and PD. All searches were conducted from inception to May 2021.The MEDLINE search was updated in February 2022 to identify additional publications. The MEDLINE search strategy is provided in “Appendix 1”. Additionally, Google Scholar was searched using the same keywords to identify other potential studies.

### Study selection process and screening

**Figure 2:**
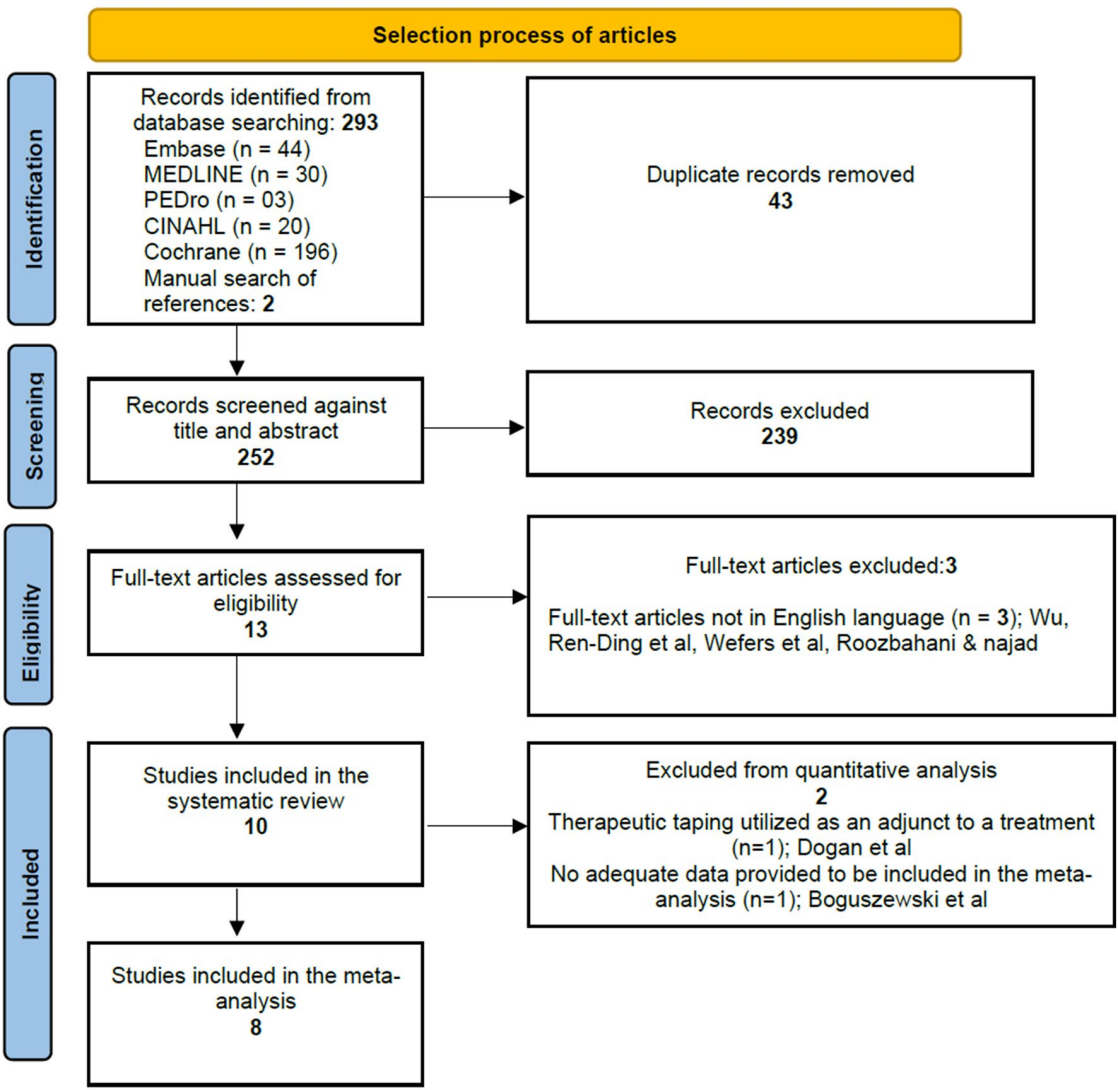
PRISMA flow diagram of search results.

All references were exported to EndNote and then transferred to Covidence for de-duplication, screening, and data extraction. Two reviewers independently screened the title and abstracts against pre-defined eligibility criteria. Full texts of the relevant abstracts were then screened by the same reviewers using the same process. Any disagreements were resolved by consensus. The number of included and excluded articles at different phases was recorded as recommended by the PRISMA guidelines and presented as a flowchart (Fig. 2).

### Data extraction

The following data were extracted from the included studies by two independent reviewers. Any disagreements were resolved through discussion or by consultation with a third reviewer. Publication details (including research title, author details, year of publication, country of investigation), details of the sample (including sample size and age of each control/intervention group), details of the intervention and comparison groups (including type and taping technique, frequency and treatment duration, outcome indicators), the significance of findings (eg: pain intensity, pain duration, anxiety and menstrual discomfort measures), and adverse events were abstracted. When the data was missing or unclear, relevant authors were contacted to obtain the necessary information.

### Data analysis

Collated evidence was summarized and presented narratively using tables and graphs. Where possible, studies with similar outcome measures and comparators were pooled together for meta-analyses. The meta-analysis was conducted using the random effect model if statistical heterogeneity, *I*^*2*^ > 50, and the mean difference (MD) was used if the studies used the same tool to measure the interested outcomes (Pain, anxiety or quality of life). Each effect was expressed at 95% of confidence interval (CI) and statistical significance at *p* < 0.05. Sensitivity analysis was conducted by attempting alternating model estimator (REM/FEM), effect size model measure (MD/ standard mean deviation (SMD)), and by excluding studies shown as outliers in funnel plots. Publication bias of included studies was assessed using funnel plots asymmetry and egger’s test. Statistical analysis was carried out in RevMan 5.4 software.

### Quality assessment

Quality of the included studies was assessed using the PEDro scale, a valid tool for risk of bias assessment of RCTs^34^. Two reviewers independently assessed the quality of included RCTs and any discrepancies were resolved by consensus. The PEDro scale consists of 11 scored items. The first item relates to the eligibility criteria and is not included in the final PEDro score. The scores from the remaining 10 items are added together to generate an overall PEDro score which is used to determine the quality of the study. Eight of these items are related to the methodological quality of the study (e.g. allocation, baseline comparability, blinding of subjects, blinding of assessors, blinding of therapists, adequate follow up and intention-to-treat). The final two items are related to statistical reporting (between group comparison, point estimates and variability)^34^. RCTs with a PEDro score between 7 and 10 were considered high quality, 4–6 were considered moderate quality and 0–3 were considered low quality^35^.

## Results

### Study selection

A total of 295 studies were identified from the database search and manual search of references. Thirteen full text articles were assessed for their eligibility. Three full texts were excluded as they were not published in English^36–38^. Ten studies were included in the systematic review. Eight studies were included in the quantitative analysis. The study selection process is presented in Fig. 2.

### Characteristics of the included studies

The included ten RCTs enrolled a total of 685 subjects. All subjects were included were aged between 13 and 35 years. Mean treatment duration and follow up period for ETT was 31.8 (1–120) days. Included studies have examined the effect of ETT compared to no intervention^19,20,31,32,39^, sham taping^19,21,31,32,39^, or other interventions, such as Pilate exercises, isometric exercises, connective tissue mobilization, Tylenol medication or auricular pressure therapy^20,31,40–42^. One study has examined effect of ETT as an adjunct to another therapy (Table 1).

**Table 1:** Characteristics and summary findings of the included studies.

Study	Country	Population (sample, age, setting)	Sample size	Intervention		Duration of treatment		Outcome indicators (tool)	Results
				Experimental (sample size)	Comparison (sample size)	Experimental	Comparison		
Pazare, 2019	India	Females with PD 18–25 years PCMC area, Pune	40	KT (20)	Isometric Exercises (20)	3 weeks (six times twice a week starting from 14 days before menstruation until its end)	8 weeks (since the third day of their menstrual cycle 5 days a week, two sessions a day, and 10 times per Session)	Pain intensity (VAS)	KT significantly improves pain compared to isometric exercises
Dogan, 2020	Turkey	Nulliparous females diagnosed with PD Over 18 years NR	60	KT + Lifestyle changes (30)	Lifestyle changes (30)	1 month (first day of the second menstrual cycle to the first day of the third menstrual cycle)	1 month (first day of the second menstrual cycle to the first day of the third menstrual cycle)	Pain intensity (VAS) Number of analgesics The Quality of life (Turkish version of the SF-36) scale	KT combined with lifestyle changes significantly improves pain reduction, quality of life compared to lifestyle changes alone
Kaur, 2017	India	Female students with complaints of PD Between 18–25 years MVP’s college of Physiotherapy, Nashik;	40	KT (20)	Connective Tissue Mobilization (20)	3 days (starts one day before menstruation)	3 days (starts one day before menstruation. The intervention consisted of 20 min sessions)	Pain intensity (NRS)_	Both KT and connective tissue mobilization are equally effective in improving pain
Boguszewski, 2020	Poland	Females with complaints of pain during menstruation NR NR	44	Elastic K-Active KT (16)	Placebo application using an inelastic tape (14) No intervention (14)	5 days	5 days 5 days	Pain intensity (VAS) Pain severity (modified version of the Laitinen questionnaire) Anxiety (STAI-X1)	Both KT and placebo application may improve menstrual pain. However, no statistically significant differences between interventions Anxiety—significantly improved with KT
Abdelaziz, 2020	Egypt	Females with complaints of pain and cramping during menstruation Between 14 to 20 years Gynecological and obstetric outpatient clinic of Eltebeen Central Hospital	60	KT (30)	Pilate exercises (30)	Three consecutive days of menstruation—one day before menstruation and would remain for approx. four to five days	12 weeks: 3 days a week, except the days of menstruation	Pain intensity (VAS) Quality of life enjoyment and satisfaction (Q-LES-Q-SF) Anxiety levels (STAI Form Y-1 and Y-2)	Both KT and Pilate exercises were effective in improvement of pain, quality of life, and anxiety Pilate exercises were superior to KT in terms of pain reduction, quality of life improvement and anxiety relief
Rodríguez, 2015	Spain	Female students who suffer from PD NR School of Medicine from the Universidad Miguel Hernández of Elche	129	A special elastic and hypoallergenic surgical tape (Cure Tape) (75)	Non-extendible meshed bandage patches (Cross Tape) (54)	4–5 days from menstruation (Until pain disappears)	4–5 days from menstruation (Until pain disappears)	Pain intensity (a 10-point scale (0 = no pain and 10 = maximum pain))	Cure tape application significantly improved pain compared to placebo application (p = 0.01)
Celenay, 2020	Turkey	Females with PD, who were nulliparous Between 18 to 35 years NR	45	KT (15)	Sham tape (15) Control group (15)	1 month (2 days a week, from the estimated day of ovulation (cycle length in days minus 14) until the next period begins)	1 month (2 days a week, from the estimated day of ovulation (cycle length in days minus 14) until the next period begins) 1 month	Pain intensity (VAS) The level of anxiety (STAI)	KT significantly improved pain intensity and anxiety compared to no application
Yum, 2017	Republic of Korea	Female students Between 13–15 years Middle School located in Seoul	125	Balance taping (33)	Medication—1 dose of Tylenol 500 mg (46) Control group (46)	Start—on the morning following the start of their period Pain intensity was measured right before the taping, as well as 1 h, 4 h, 8 hours, and 24 h after	The medication group took only 1 dose of Tylenol 500 mg, but midterm and final exam periods were made an exception Pain intensity was measured right before the taping, as well as 1 h, 4 h, 8 h, and 24 h after	Pain intensity (VAS)	Balance taping significantly improved pain compared to medications
Lim, 2013	Korea	Unmarried, non- parous females without pathologic findings in the pelvic cavity, whose menstrual pain scores were five or higher on a visual analogue scale (VAS) In their twenties and thirties NR	34	KT (11)	Spiral taping (10) Control group (13)	Three weeks—total six times (twice a week starting from 14 days before menstruation until its end)	Three weeks—total six times (twice a week starting from 14 days before menstruation until its end) Three weeks	Pain intensity (VAS)	Both KT and spiral taping (*p* < 0.05) significantly improved pain relief. KT was more effective in pain relief
Mejías-Gil, 2021	Spain	PD grade 2 and 3 of Andersch and Milsom classification Between 18 and 30 years Women enrolled in the University of Extremadura	108	KT (22)	Placebo KT application (21) Auricular pressure (21) Placebo auricular pressure (22) Control group (22)	From initial 4 h to 72 h of the menstruation cycle	From initial 4 h to 72 h of the menstruation cycle	Pain intensity (VAS)	Both KT and auricular acupressure have a beneficial effect on pain relief in women with primary dysmenorrhea

*KT* kinesiotaping, *PCMC* pimpri-chinchwad municipal corporation, *PD* primary dysmenorrhea, *Q-LES-Q-SF* quality of life enjoyment and satisfaction, *NR* not reported, *NRS* numerical rating scale, *STAI* spielberger state-trait anxiety inventory, *ST* sham taping, *VAS* visual analogue scale.

### Quality evaluation

**Figure 3:**
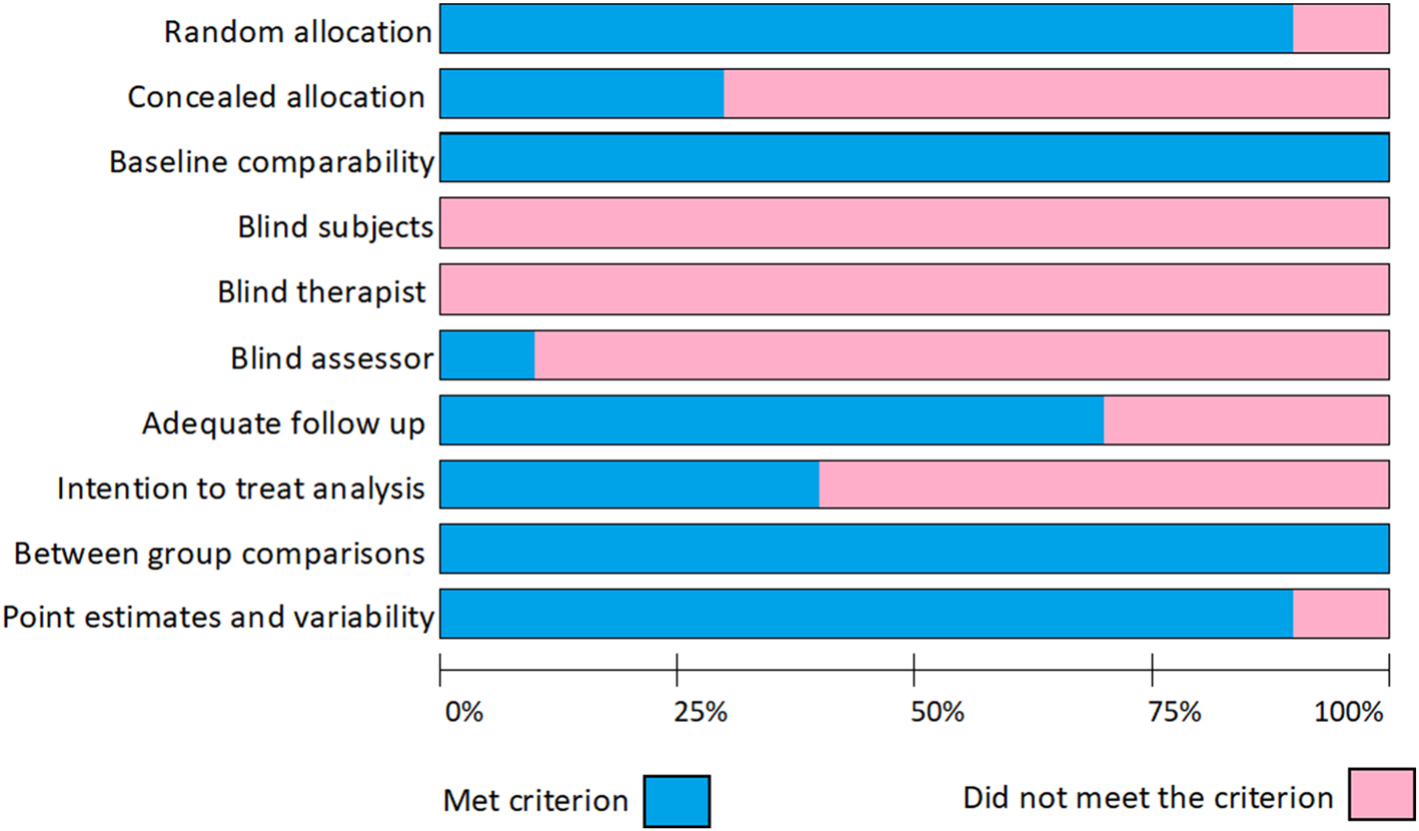
Risk-of-bias of the included studies, presented as the percentage that met the PEDro scale criteria. Abbreviation: *PEDro* physiotherapy evidence database.

The PEDro score of the included studies ranged from 4 to 7 with a median score of 5 (moderate quality) (Table 2). All the studies satisfied the baseline comparability and between group comparison criteria. However, no study met the criteria for subject or therapist blinding, presumably due to the nature of the therapy which could not be blinded (Fig. 3).

**Table 2:** Risk of bias evaluation of included studies.

Study	PEDro scale item											PEDro
	1*	2	3	4	5	6	7	8	9	10	11	Score
Abdelaziz 2020	Y	Y	Y	Y	N	N	N	Y	Y	Y	Y	7
Boguszewski 2020	N	Y	N	Y	N	N	N	N	N	Y	Y	4
Celenay 2020	N	Y	N	Y	N	N	N	Y	N	Y	Y	5
Dogan 2020	Y	Y	Y	Y	N	N	Y	Y	N	Y	Y	7
Kaur 2017	Y	Y	N	Y	N	N	N	Y	Y	Y	N	5
Pazare 2019	Y	Y	N	Y	N	N	N	N	N	Y	Y	4
Rodríguez 2015	Y	Y	N	Y	N	N	N	N	Y	Y	Y	5
Lim 2013	N	Y	N	Y	N	N	N	Y	N	Y	Y	5
Yum 2017	Y	N	N	Y	N	N	N	Y	N	Y	Y	4
Gil 2021	Y	Y	Y	Y	N	N	N	Y	Y	Y	Y	7

Y: yes N: no *: Not considered for total score.

1: Eligibility criteria 2: Random allocation 3: Concealed allocation 4: Baseline comparability 5: Blind subjects 6: Blind therapist 7: Blind assessor 8: Adequate follow up 9: Intention to treat analysis 10: Between group comparisons 11: Point estimates and variability.

### Pain intensity: ETT versus no intervention

**Figure 4:**
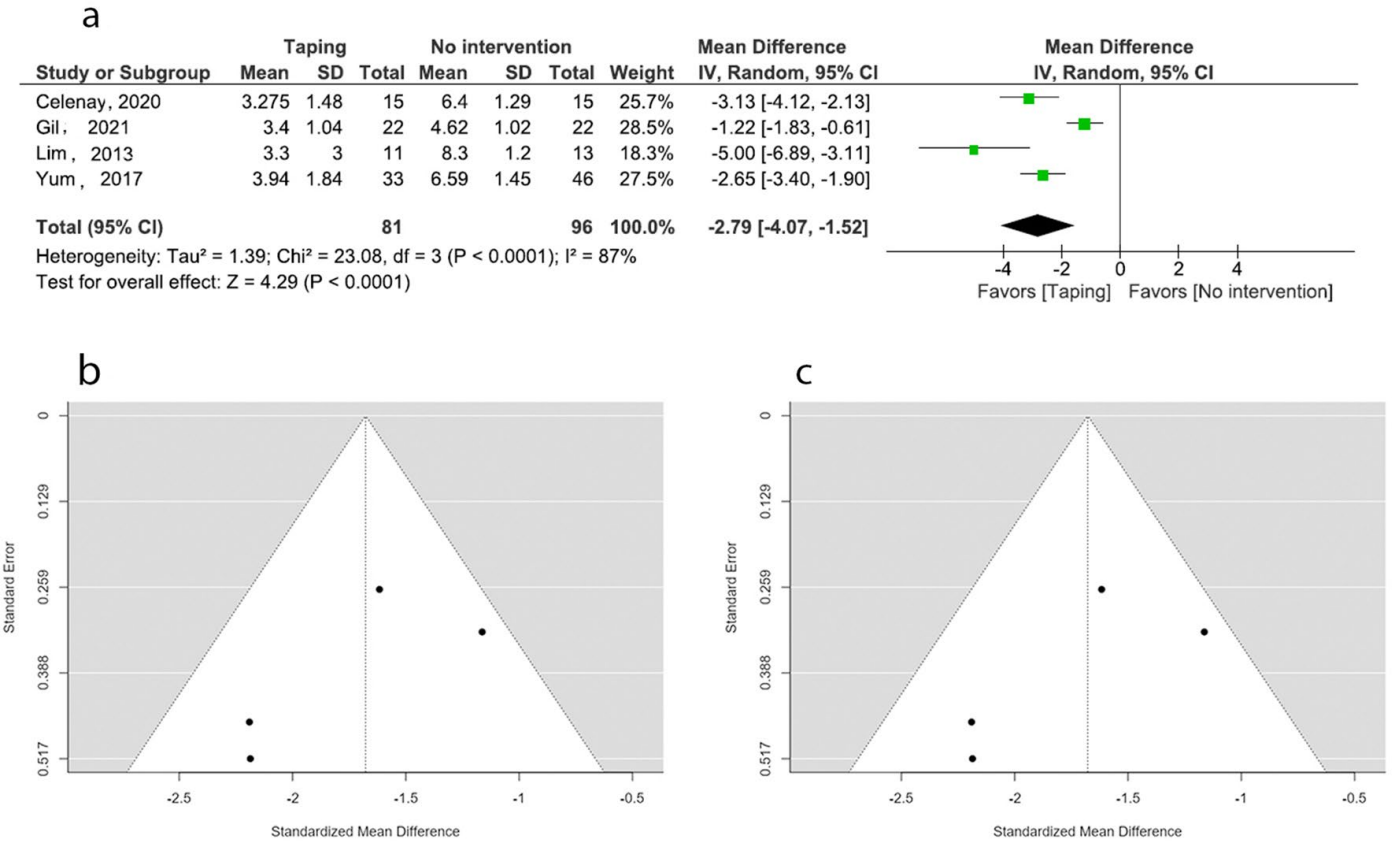
Meta-analysis and sensitivity analysis (effect of ETT vs no intervention on pain). (**a**) Forest plot (REM/MD). (**b**) Funnel plot (REM/MD). (**c**) Funnel plot (REM/SMD).

Five studies examined the effect of ETT on pain intensity in PD compared to no intervention^19,20,31,32,39^. Of these, four studies (n = 177 females with PD) were included in the meta-analyses^20,31,32,39^. Random effect model and mean difference (MD) were utilized in the meta-analysis. Studies with moderate level of quality suggested an overall estimate of − 2.79 (95% CI: − 4.07, − 1.52) favoring ETT over no intervention. Heterogeneity was high (*I*^*2*^ = 87%). All four studies favored ETT with a raw effect size ranging from − 1.22 to − 5.0 on the Visual Analogue Scale (VAS). Sensitivity analysis was conducted due to the high heterogeneity in meta-analysis by attempting standard mean difference (SMD) instead of MD. This produced a lesser overall estimate of − 1.67 (95% CI: − 2.12, − 1.22) with a reduction of heterogeneity (*I*^*2*^ = 33%). Further sensitivity analysis was attempted by the fixed effect model with SMD. The fixed model produced a slighter lower overall estimate of − 1.63 (95% CI: − 1.98, − 1.28) with a similar heterogeneity (*I*^*2*^ = 33%) and significant overall effect (Fig. 4).

### Pain intensity: ETT versus sham taping

**Figure 5:**
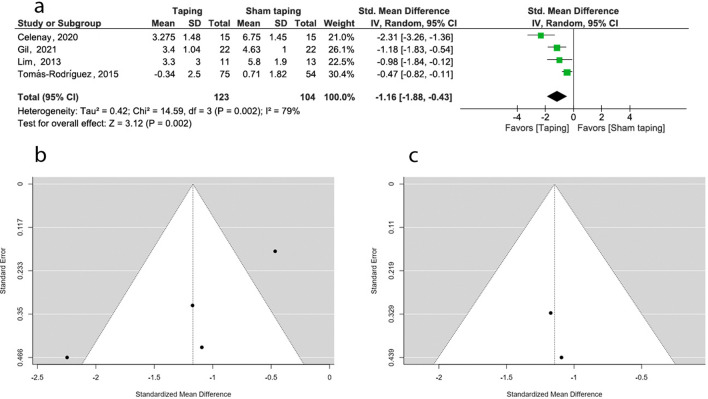
Meta-analysis and sensitivity analysis (effect of ETT vs other Sham taping on pain). (**a**) Forest plot (REM/SMD). (**b**) Funnel plot (REM/SMD). (**c**) Funnel plot (FEM/SMD, excluding two outliers).

Five studies examined the effect of ETT on pain intensity in PD compared to sham taping^19,21,31,32,39^. Of these, four (n = 277 females with PD) were included in the meta-analyses. Random effect model and SMD were attempted for the meta- analysis^21,31,32,39^. Studies with moderate level of quality suggested an overall estimate of − 1.16 (95% CI: − 1.88, − 0.43) favoring ETT over sham taping. Heterogeneity was high (*I*^*2*^ = 79%). All the four studies favored ETT with raw effect size ranging from − 0.47 to − 2.31 on the VAS scale. Sensitivity analysis was conducted due to the high heterogeneity by attempting the fixed effect model. The fixed model produced a lower overall estimate of − 0.81 (95% CI: − 1.09, − 0.53) with a similar heterogeneity (*I*^*2*^ = 79%). Further sensitivity analysis was carried out by excluding the outliers^21,32^. This eliminated the heterogeneity (*I*^*2*^ = 0%) with a higher overall estimate of − 1.11 (95% CI: − 1.63, − 0.59) and the overall effect was significant (Fig. 5).

### Pain intensity: ETT versus other interventions

**Figure 6:**
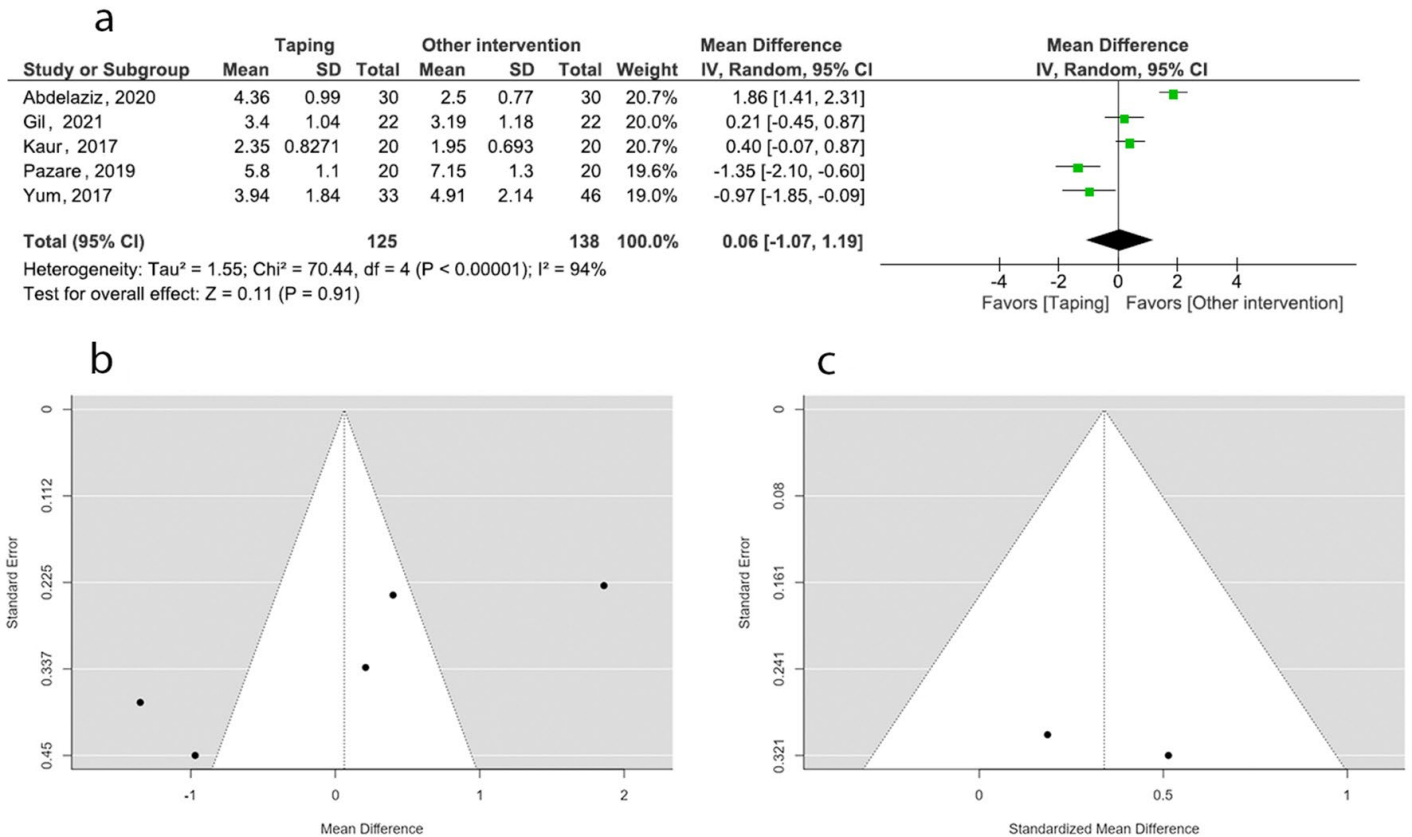
Meta-analysis and sensitivity analysis (effect of ETT vs other interventions on pain). (**a**) Forest plot (REM/MD). (**b**) Funnel plot (REM/MD). (**c**) Funnel plot (FEM/SMD, excluding three outliers).

Five studies including 263 patients, examined the effect of ETT compared to other therapeutic interventions on pain intensity in PD^20,31,40–42^, and all five were included in the meta-analyses. Random effect model and mean difference (MD) were attempted for the meta- analysis. Studies with moderate level of quality suggested that there is no significant difference between ETT and other interventions (pooled MD = 0.06, 95% CI: − 1.07, 1.19). Heterogeneity was high (*I*^*2*^ = 94%). Two studies favored ETT with raw effect sizes of − 0.97 and − 1.35. Three studies favored the other interventions over ETT. Sensitivity analysis was conducted due to the high heterogeneity by attempting SMD instead MD. This attempt produced a higher overall estimate of 0.24 (95% CI: − 0.76, 1.23) without having any significant difference between ETT and other interventions. There was a comparably small effect on heterogeneity (*I*^*2*^ = 93%). Then fixed model attempted with SMD produced a higher overall estimate of 0.15 (95% CI: − 0.11, 0.41) without having any significant difference between ETT and other interventions. There was no significant effect on heterogeneity (*I*^*2*^ = 93%). Further sensitivity analysis was carried out excluding outliers in the plot^20,40,41^. This produced a higher overall estimate of 0.34 (95% CI: − 0.09, 0.77) with eliminating heterogeneity (*I*^*2*^ = 0%). Overall effect was insignificant. (Fig. 6).

### Anxiety: ETT versus no intervention

**Figure 7:**
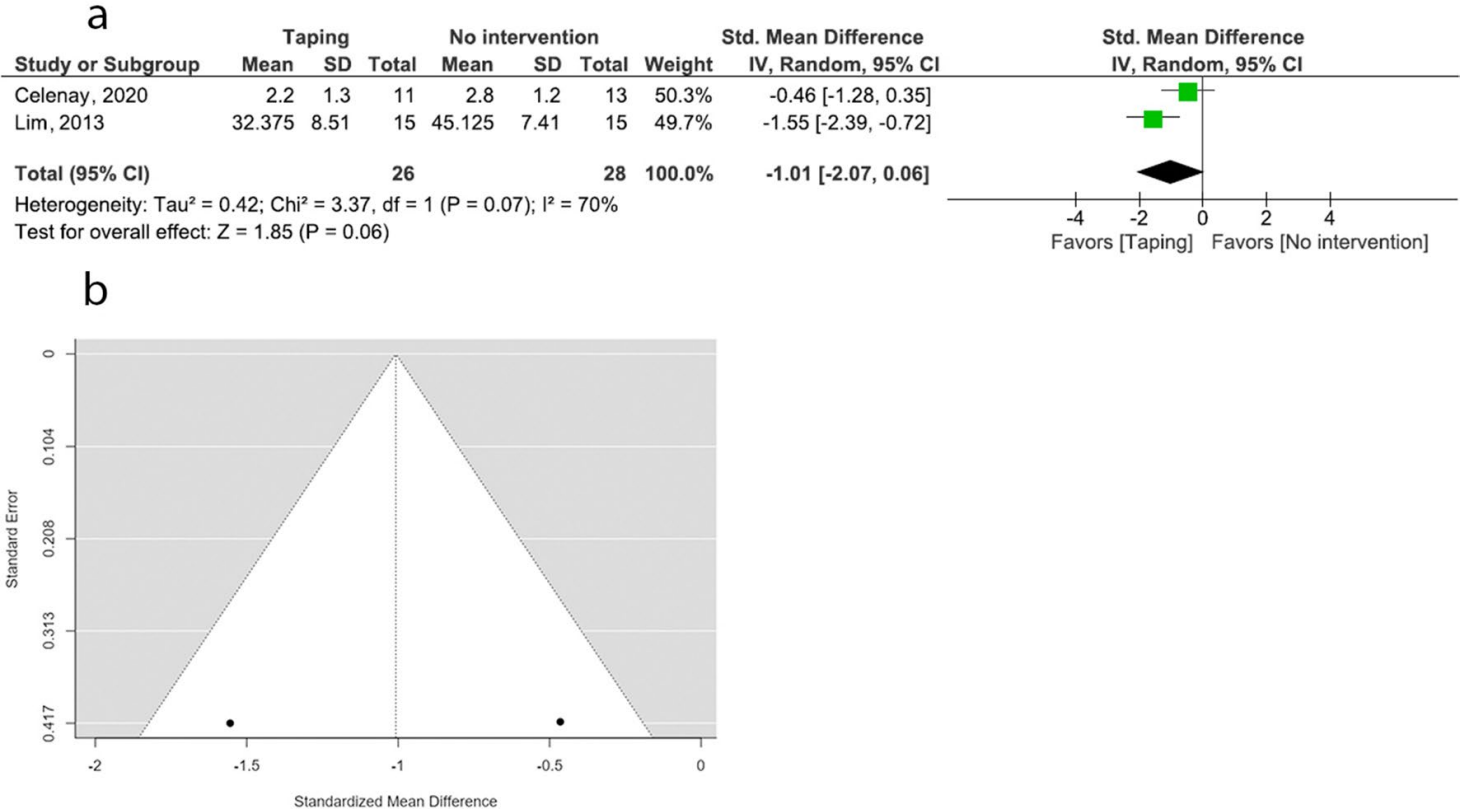
Meta-analysis and sensitivity analysis (effect ETT vs no interventions on anxiety). (**a**) Forest plot (REM/MD). (**b**) Funnel plot (REM/MD). (**c**). Funnel plot (FEM/SMD, excluding three outliers).

Four studies examined the effect of ETT on anxiety^19,32,39,40^. One study examined the effect of ETT compared to Pilates exercises^40^. Both ETT and Pilates exercises significantly improved anxiety among patients with PD (*p* < 0.001). However, the effect of Pilates exercises was superior to the ETT application (*p* < 0.001). Three studies examined the effect of ETT on anxiety compared to no intervention^19,32,39^. One study did not provide adequate data (post interventional variance measure) to be pooled in a meta-analysis^19^ although the study indicates a significant improvement of anxiety with ETT in PD. Two studies were pooled together to quantitatively assess the effect of ETT on anxiety assessed by Spielberger State-Trait Anxiety Inventory (STAI) and anxiety subset of Menstrual Distress Questionnaire (MDQ). Random effect model and standard mean difference (SMD) were attempted for the meta- analysis. Moderate quality RCTs suggested that ETT is effective in improving anxiety compared to no intervention (pooled SMD = − 1.01, 95% CI: − 2.07, 0.06). Heterogeneity was high (*I*^*2*^ = 70%). Both studies favored ETT over no intervention. Sensitivity analysis was conducted due to the high heterogeneity by attempting the fixed effect model. The fixed model produced a higher overall estimate of − 1.00 (CI: − 1.58, − 0.42) favoring ETT over no intervention with similar heterogeneity (*I*^*2*^ = 70%). There was a significant overall effect (Fig. 7).

### Quality of life

Two high quality RCTs examined the effect of ETT on the quality of life in women with PD^40,41^. One study found that ETT significantly improved quality of life among patients with PD^40^ (*p* < 0.001). Another study compared the effect of ETT as an adjunct to lifestyle changes on quality of life and indicates that the ETT as an adjunct significantly improves quality of life when compared to lifestyle changes alone (*p* < 0.05)^41^. Studies could not be meaningfully pooled due to their difference of the study design.

### Adverse events

Three RCTs of moderate to high-quality investigated potential adverse effects of taping^20,32,41^. Two studies (n = 60) where participants were treated with taping reported no adverse events^32,41^. One study (n = 33) reported that two participants experienced allergic skin reactions, and one-person experienced dizziness as adverse reactions^20^. Overall adverse events reported among participants is only 3.22%.

### Publication bias

The publication bias of the three meta-analyses was assessed by funnel plots. The funnel plots constructed for the effect of ETT versus no intervention on pain (*p* = 0.002) (Fig. 4b), ETT versus sham taping on pain (*p* < 0.001) (Fig. 5b), and ETT versus other interventions on pain (*p* = 0.004) (Fig. 6b), are asymmetrical according to Egger’s test indicating a possible risk of bias. However, the funnel plot constructed for the effect of ETT versus no intervention on pain improved its symmetry when meta-analysis conducted with SMD (*p* = 0.186) (Fig. 4c). Funnel plots constructed for the effect ETT versus sham taping on pain (*p* = 0.884) (Fig. 5c), and ETT versus other intervention on pain improved their symmetry when the meta-analysis was conducted with the outliers removed (*p* = 0.457) (Fig. 6c). The funnel plot constructed for the effect of ETT versus no intervention on anxiety is symmetrical suggesting lower likelihood of publication bias (*p* = 0.064) (Fig. 7b).

### Evidence summary

Favorable evidence was found for the effect of ETT on quality-of-life outcomes with the evidence being of high-quality. Favorable evidence was found on the effect of ETT on pain intensity and anxiety with the evidence being of moderate quality (Table 3).

**Table 3:** Evidence summary.

Outcome indicator	Type of therapeutic taping	Quality of evidence		
		Weak	Moderate	High
Pain intensity ^19,21,31,32,39^	ETT		X	
Anxiety^32,39^	ETT		X	
Quality of life ^40,41^	ETT			X

*ETT* elastic therapeutic taping.

## Discussion

This current systematic review and meta-analysis aimed to investigate whether taping is an effective treatment for PD in improving pain, anxiety, and quality of life. The summarized findings of the review indicate evidence exists to support therapeutic taping in improving pain intensity, anxiety, and quality of life. Further, with a moderate quality of evidence, our meta-analyses confirmed that ETT is an effective therapeutic application in improving pain of PD compared to no intervention and placebo application. Furthermore, the moderate quality evidence in the meta-analysis indicates that effect of ETT on pain is not significantly different from other interventions included in this review. Additionally, with moderate quality evidence, our meta-analysis indicates that the ETT is effective in improving anxiety associated with PD. Though we found the adverse effects of skin allergies and dizziness associated with therapeutic taping, they are minor and are no worse that of the use of medications, such as NSAIDs^42^. A previous systematic review which assessed the efficacy of physiotherapy treatment for PD indicated that KT is an effective option in improving pain, anxiety, and several menstrual complaints^43^, supports the current findings. However, the current review includes additional nine studies which were not included in the previous review^43^.There are no other studies that the authors are aware of, that explore the safety measures related to therapeutic taping application for PD.

Abnormal increases of prostaglandin and vasopressin have been identified as the possible cause of PD^44^. This abnormal increase of uterine hormones is known to shrink the uterus and thereby reduce blood and oxygen supply which may cause pain^44^. ETT applied on skin may induce underlying muscle contractions and relaxations which would improve uterine blood flow^45^. It is hypothesized that the pain inhibition attributed to tension generated from ETT stimulates afferent nerve fibers and facilitates pain inhibitory mechanisms^46^. Most of the included studies used KT as the therapeutic taping application in the current review. The potential pain reduction mechanism of KT application may include producing sensory tactile impulses on the skin that are able to block or reduce the arrival of pain sensations to the brain^47^. Also, KT application may increase blood flow by microscopically lifting the skin from the facia and activating the skin—organ reflex^48–50^.

This is the first systematic review and meta-analysis to assess the effects of therapeutic taping for PD. The strengths of this review include the comprehensive search strategy and the eligibility criteria used to retrieve studies which used all types of therapeutic taping applications. All included studies used validated outcome measures of pain, anxiety, and quality of life such as VAS, Numerical Rating Scale (NRS), STAI and Quality of Life Enjoyment and Satisfaction (Q-LES-Q-SF)^51–53^. The current systematic review provides a rigorous summary of the current evidence related to the therapeutic taping usage for PD. Additionally, this review provides directions to conduct future RCTs with a higher quality to evaluate the safety and effectiveness of therapeutic taping application for PD. Studies which used sham, or no intervention control groups provide useful information on the natural regression of clinical symptoms and placebo effect due to treatment expectations.

Some limitations must be acknowledged. Studies were only included if they were published in English, potentially limiting the inclusion of all relevant research on this topic. Variations of clinical attributes such as treatment duration, age category, tape application method, and follow-up period might have contributed to the substantial heterogeneity in pooled estimates. Due to the small number of included studies subgroup analysis or meta-regression could not be performed to explore the sources of heterogeneity. Hence the results of this systematic review and meta-analysis should be interpreted cautiously. Most RCTS included an average short term follow up of 31.8 days (1–120), hence the systematic review findings can be generalized to interpret the short-term efficacy of therapeutic taping at least for the first menstrual cycle with ETT application for PD. Only three out of ten studies^20,32,41^ have investigated the adverse reactions related to therapeutic taping, other studies did not describe the safety aspects of therapeutic taping application.

Future RCTs with improved methodological quality by considering the allocation concealment and blinding to minimise possible biases, are suggested. Further studies with appropriate study designs are necessary to determine the efficacy and safety of therapeutic taping as an adjunct to other interventions. RCTs with longer follow-up duration should be conducted to determine the long-term effects of therapeutic taping on PD.

In conclusion, this systematic review and meta-analysis provides moderate to high quality evidence indicating the relative safety and efficacy of ETT approach in treating pain and anxiety associated with PD in the short term. Additionally, it supports the effect of ETT in improving quality of life among females with PD. This conclusion should be verified through longer, high-quality RCTs with larger sample sizes. Future RCTs should be designed with a better methodological quality and long-term follow-up to establish a firm conclusion on the usage of therapeutic taping for PD.

## Data Availability

The datasets generated and/or analyzed during the current systematic review are available from the corresponding author upon a reasonable request.

## Appendix 1: MEDLINE

**Table:**

#	Searches	Results
1	Kinesiotap*.mp	189
2	Kinesio tap*.mp	562
3	Exp Athletic Tape/	750
4	Elastic tap*.mp	120
5	Kinesiology Tap*.mp	189
6	KT tap*.mp	8
7	k-tap*.mp	18
8	Taping*.mp	2251
9	Kinematic tap*.mp	1
10	Medical tap*.mp	53
11	Balance tap*.mp	16
12	Elastic therapeutic tap*.mp	37
13	Kinesiology therapeutic tap*.mp	0
14	Therapeutic tap*.mp	80
15	Tape.mp	22,075
16	1 or 2 or 3 or 4 or 5 or 6 or 7 or 8 or 9 or 10 or 11 or 12 or 13 or 14 or 15	23,318
17	Exp dysmenorrhea/	4298
18	Dysmenorrh*.tw	6509
19	Primary dysmenorrh*.mp	1321
20	Premenstrual syndrome/	4070
21	(Period* adj4 pain*).mp	5037
22	(Period* adj4 menstruat*).mp	245
23	(Menstruat* adj3 cramp*).mp	17
24	(Menstruat* adj3 discomfort*).mp	26
25	(Menstruat* adj3 symptom*).mp	237
26	17 or 18 or 19 or 20 or 21 or 22 or 23 or 24 or 25	16,669
27	16 and 26	30
